# Highly selective whole-cell 25-hydroxyvitamin D_3_ synthesis using molybdenum-dependent C25-steroid dehydrogenase and cyclodextrin recycling

**DOI:** 10.1186/s12934-024-02303-6

**Published:** 2024-01-20

**Authors:** Dennis Kosian, Max Willistein, Ralf Weßbecher, Constantin Eggers, Oliver May, Matthias Boll

**Affiliations:** 1 Faculty of Biology – Microbiology, University of Freiburg, 79104 Freiburg, Germany; 2 DSM Nutritional Products, Koninklijke DSM N.V., Kaiseraugst, 4303 Switzerland

**Keywords:** Vitamin D_3_, 25-hydroxyvitamin D_3_, Calcifediol, Calcitriol, Whole-cell biocatalysis, *Thauera aromatica*, Molybdenum-dependent hydroxylase, Steroid C25 hydroxylase, Cyclodextrin

## Abstract

**Background:**

The global prevalence of vitamin D (VitD) deficiency associated with numerous acute and chronic diseases has led to strategies to improve the VitD status through dietary intake of VitD-fortified foods and VitD supplementation. In this context, the circulating form of VitD_3_ (cholecalciferol) in the human body, 25-hydroxy-VitD_3_ (calcifediol, 25OHVitD_3_), has a much higher efficacy in improving the VitD status, which has motivated researchers to develop methods for its effective and sustainable synthesis. Conventional monooxygenase-/peroxygenase-based biocatalytic platforms for the conversion of VitD_3_ to value-added 25OHVitD_3_ are generally limited by a low selectivity and yield, costly reliance on cyclodextrins and electron donor systems, or by the use of toxic co-substrates.

**Results:**

In this study, we used a whole-cell approach for biocatalytic 25OHVitD_3_ synthesis, in which a molybdenum-dependent steroid C25 dehydrogenase was produced in the denitrifying bacterium *Thauera aromatica* under semi-aerobic conditions, where the activity of the enzyme remained stable. This enzyme uses water as a highly selective VitD_3_ hydroxylating agent and is independent of an electron donor system. High density suspensions of resting cells producing steroid C25 dehydrogenase catalysed the conversion of VitD_3_ to 25OHVitD_3_ using either O_2_ via the endogenous respiratory chain or externally added ferricyanide as low cost electron acceptor. The maximum 25OHVitD_3_ titer achieved was 1.85 g L^–1^ within 50 h with a yield of 99%, which is 2.2 times higher than the highest reported value obtained with previous biocatalytic systems. In addition, we developed a simple method for the recycling of the costly VitD_3_ solubiliser cyclodextrin, which could be reused for 10 reaction cycles without a significant loss of quality or quantity.

**Conclusions:**

The established steroid C25 dehydrogenase-based whole-cell system for the value-adding conversion of VitD_3_ to 25OHVitD_3_ offers a number of advantages in comparison to conventional oxygenase-/peroxygenase-based systems including its high selectivity, independence from an electron donor system, and the higher product titer and yield. Together with the established cyclodextrin recycling procedure, the established system provides an attractive platform for large-scale 25OHVitD_3_ synthesis.

**Supplementary Information:**

The online version contains supplementary material available at 10.1186/s12934-024-02303-6.

## Background

Vitamin D (VitD) is well known for its role in calcium and phosphate homeostasis in the human body, and a deficiency of the prohormone is primarily associated with skeletal diseases such as rickets [1]. There is now growing evidence linking VitD deficiency to cardiovascular disease, some cancers, neurological disorders, autoimmune diseases (e.g., multiple sclerosis, type 2 diabetes) and pulmonary diseases [2–4]. Recent studies have investigated the role of VitD in the context of the COVID-19 pandemic and have shown a strong correlation between low serum levels of VitD and the risk for and severity of COVID-19 infections [5, 6].

**Fig. 1:**
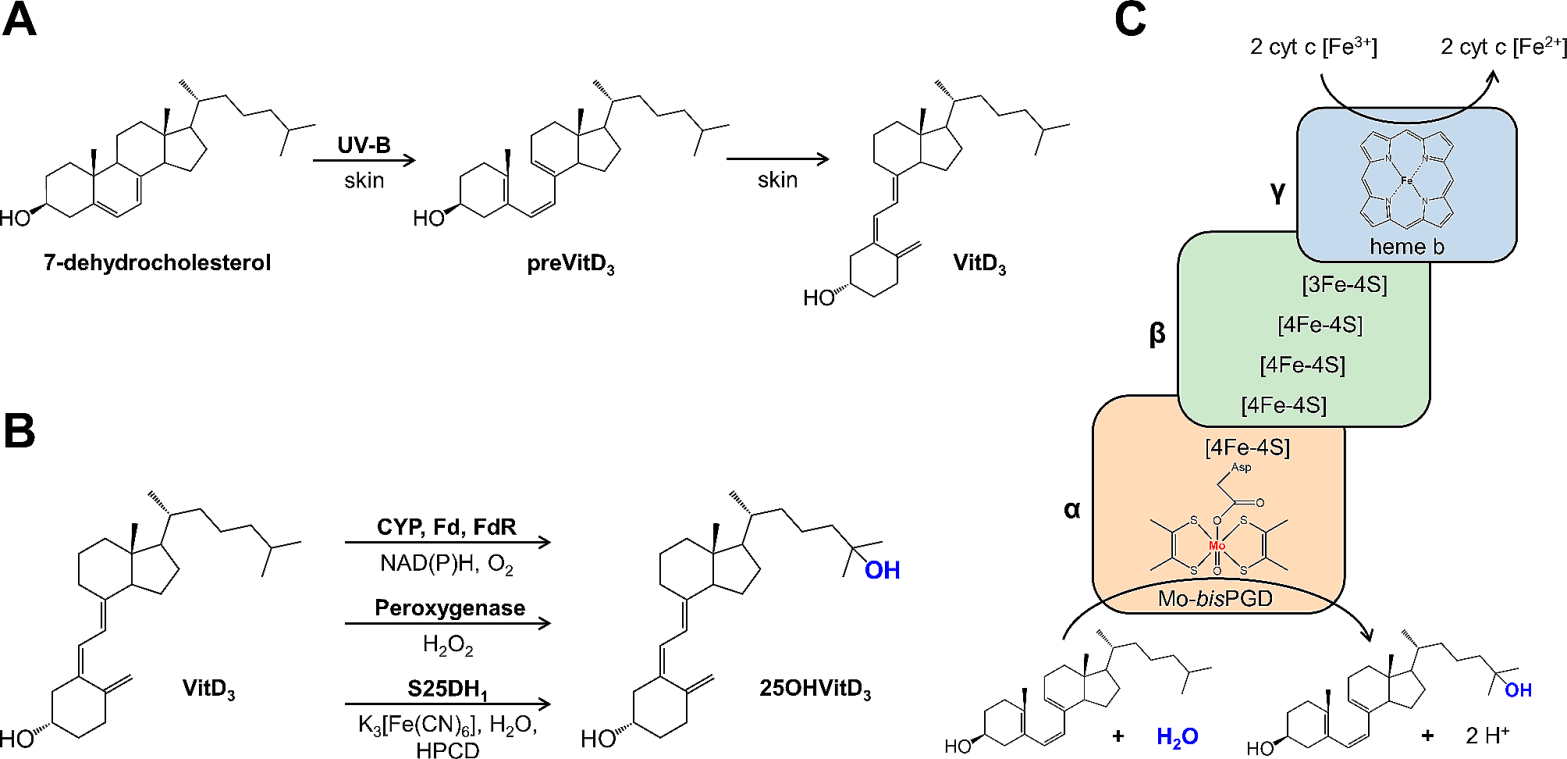
Synthesis of VitD_3_ in the human skin (**A**), hydroxylation to 25OHVitD_3_ by different enzymes (**B**), subunit composition and reaction catalysed by S25DH_1_ from *Sterolibacterium denitrificans* (**C**)

There are two forms of VitD: the VitD_2_, found in UV-irradiated fungi or yeast, and the VitD_3_ (cholecalciferol). The latter is synthesised in the human skin from endogenous 7-dehydrocholesterol after exposure to solar UV-B radiation to preVitD_3_ followed by thermal isomerisation (Fig. 1A). In the liver, several cytochrome P450 enzymes (CYPs) hydroxylate VitD_3_ to 25-hydroxy-VitD_3_ (calcifediol, 25OHVitD_3_), the clinically relevant, circulating form of VitD_3_ (Fig. 1B). Finally, the active hormone calcitriol (1α,25(OH)_2_VitD_3_) is formed by a second CYP-dependent hydroxylation in the kidneys [2, 3].

There is a global prevalence of VitD deficiency, which has been associated with numerous acute and chronic diseases [2, 3]. The level of physiological 25OHVitD serves as an indicator of VitD deficiency, with serum concentrations below 20 µg L^–1^ (50 nmol L^–1^) representing a threshold. Using this definition, around 40% of the European population is estimated to be VitD deficient. Strategies to improve the VitD status include dietary intake of VitD-fortified foods and supplementation [2]. Supplementation with 25OHVitD_3_ has a number of advantages in comparison to VitD_3_/VitD_2_: (i) serum 25OHVitD_3_ levels are increased more rapidly; (ii) 25OHVitD_3_ has a linear dose-response curve; (iii) there is no dependence on liver CYPs, which is beneficial in patients with impaired hepatic function; (iv) the significantly higher water solubility of 25OHVitD_3_ reduces its accumulation in adipose tissue and improves its uptake in patients with malabsorption [7–10]. In summary, 25OHVitD_3_ is considered to be 3- to 6-fold more potent than VitD_3_/VitD_2_, which is particularly beneficial when an immediate increase in serum 25OHVitD_3_ is required.

Due to the high efficiency of 25OHVitD_3_ supplements in improving human VitD status, there is a high demand for effective methods to synthesise it from readily available precursors. This demand is further increased by the use of 25OHVitD_3_ as effective feed additive for poultry or pig nutrition [11–13]. The chemical synthesis of 25OHVitD_3_ from steroid precursors, e.g. produced in yeast, still plays an important role in industrial processes and proceeds via 25-OH-7-dehydrocholesterol followed by UV irradiation and thermal isomerization to the product [14, 15]. However, the rather low yields and selectivity, especially with respect to the hydroxylation at C25, have motivated researchers to explore one-step enzymatic systems that directly convert VitD_3_ to the value-added 25OHVitD_3_. Over the past two decades, a number of studies have evaluated numerous CYPs from vertebrate liver, fungi or bacteria for the O_2_-dependent hydroxylation of VitD_3_ to 25OHVitD_3_ ( [16], Table 1 and references therein, Fig. 1B). Since CYPs consume the electron donor NAD(P)H stoichiometrically, the costly regeneration of NAD(P)H can be facilitated by using whole-cell systems expressing CYPs.

To date, the highest titer of 25OHVitD_3_ synthesised from VitD_3_ via a whole-cell system involving CYPs is 830 mg L^–1^ after 60 h with a yield of 42%, as reported for *Bacillus cereus* zju 4-2 [17] (Table 1). A higher yield (74.4%) and productivity (287 mg L^–1^ h^–1^) has been reported for a recombinant *Rhodococcus erythropolis* expressing a CYP that converts VitD_3_ to 25OHVitD_3_ [18]. Besides the problem of incomplete substrate conversion, a major disadvantage of CYP enzymes is their rather low specificity, often leading to the formation of undesired by-products requiring expensive HPLC-based purification protocols. In particular, the formation of the highly potent hormone calcitriol after a second hydroxylation at C1 is problematic. As an alternative, fungal peroxygenases have been used for the hydroxylation of VitD_3_ to 25OHVitD_3_, using H_2_O_2_ instead of O_2_ + NAD(P)H as co-substrate [19, 20] (Fig. 1B). However, peroxygenase-dependent systems are typically used in vitro due to their toxic co-substrate.

**Table 1:** Comparison of selected whole-cell based production systems of 25OHVitD_3_

Organism	Enzyme	VitD_3_ (mg L^–1^)	Additives	25OHVitD_3_ (mg L^–1^)	Time	Ref.
*Escherichia coli* BL21 (DE3)	recombinant CYP105A1	100	dimethylsulfoxid, 1% (v/v)	2.5	24 h	[21]
*Kutzneria albida*	CYP	500	HPCD, 1% (w/v)	70.4	48 h	[22]
*Bacillus megaterium*	recombinant CYP109A2 (T103A mutant)	577	HPCD, 2.25% (w/v) saponin, 0.0825% (w/v)	282.7	48 h	[23]
*Pseudonocardia* sp. KCTC 1029BP	n.d.	600	ethanol, 0.2% (v/v) HPCD, 0.1% (w/v)	356	120 h	[24]
*Rhodococcus erythropolis*	recombinant CYP (T107A mutant)	769	partially methylated β-cyclodextrin, 1.5% (w/v)	573	2 h	[18]
*Bacillus cereus* zju 4-2	n.d.	2000	propylene glycol, 9% (v/v) ethanol, 1% (v/v)	830	60 h	[17]
*Thauera aromatica* K172	recombinant S25DH_1_	1780	HPCD, 12.5% (w/v) ethanol, 5% (v/v)	1850	50 h	this work

Steroid C25 dehydrogenases (S25DHs) have been identified in facultatively anaerobic bacteria that use side-chain containing steroids as a carbon and energy source under denitrifying conditions, and hydroxylate their substrate to C25 tertiary alcohols [25, 26]. These enzymes with moderate oxygen sensitivity (50% loss of activity in crude extracts after 24 h) belong to the dimethylsulfoxide (DMSO) reductase family of molybdenum cofactor (Moco) containing enzymes, are composed of three subunits, and use water as hydroxylating agent (Fig. 1C). A periplasmic S25DH (designated S25DH_1_) has originally been isolated from the Gram-negative β-proteobacterium *Sterolibacterium denitrificans* and most likely uses cytochrome c as in vivo electron acceptor, which can be replaced by the artificial ferricyanide (K_3_[Fe(CN)_6_]) in vitro [25]. Catalysis takes place at Moco (molybdo-*bis*-pyranopterin guanine dinucleotide, Mo-*bis*PGD) bound to the α-subunit, while the β- (FeS cluster cofactors), and γ-subunits (heme b cofactor) are involved in electron transfer from the substrate to the external acceptor. A fourth gene product (δ-‘subunit’) serves as an essential chaperone for proper folding and cofactor insertion. The proposed mechanism of S25DHs involves hydride abstraction from tertiary C25 to the oxidised Mo(VI) = O yielding Mo(IV)–OH and a carbocation intermediate, followed by the transfer of the hydroxyl group back to C25 of the substrate [27]. In addition to the natural substrate, cholest-4-en-3-one, S25DH_1_ from *S. denitrificans* converts VitD_3_ to 25OHVitD_3_ with an apparent selectivity of 100% in the presence of 2-hydroxypropyl-β-cyclodextrin (HPCD) and K_3_[Fe(CN)_6_] as acceptor [28]. Recently, we established a heterologous production platform for the three structural subunits and the chaperone in the denitrifying β-proteobacterium *Thauera aromatica* K172. Heterologous overproduction of the S25DH_1_ was achieved using an isopropyl β-D-1-thiogalactopyranoside (IPTG) induced *tac* promotor in the presence of gentamycin, resulting in a 6.5-fold higher specific activity in the soluble cell extract of. *T. aromatica* than in that of the wild type reaching 2.9 nmol min^–1^ (mg protein)^–1^ [29].

Based on the established heterologous production platform for S25DHs in *T. aromatica*, we aimed to establish a whole-cell platform for the specific conversion of VitD_3_ to 25OHVitD_3_. Using resting *T. aromatica* cells expressing the genes encoding S25DH_1_ and the chaperone, we achieved a product titer 2.2-fold higher than the highest reported for CYP-dependent whole-cell systems, with a selectivity > 99% and a substrate conversion > 99%. S25DH_1_ was surprisingly oxygen tolerant in whole cells, allowing VitD_3_ hydroxylation to be linked to the endogenous aerobic respiratory chain. Finally, an efficient procedure for HPCD recycling was established which is essential for the implementation of the whole-cell system for potential applications.

## Methods

### Cultivation of S25DH_1_-producing *T. aromatica* K172

Cultivation was performed at 30 °C under fully aerobic, fully anaerobic or semi-aerobic conditions (static bottle with no active oxygen removal) in mineral salt medium at a 1 or 2 L scale as described [30]. Sodium acetate (15 mM) served as electron donor and carbon source, NaNO_3_ (15 mM) or O_2_ as electron acceptors. The pH was adjusted to 8.0. Gentamycin was added to a final concentration of 50 µg mL^–1^. Supplementation with vitamins (VL-7 stock solution), trace elements (SL-10 stock solution) and MgSO_4_/CaCl_2_ stock solutions was as described [30].

Growth was monitored by optical density measurements at 578 nm (OD_578 nm_). Nitrate concentration (Quantofix nitrate/nitrite II, Macherey-Nagel, Düren, Germany) and pH (pH-Fix 4.5–10, Roth, Karlsruhe, Germany) were determined. The medium was supplemented with sodium acetate/acetic acid (depending on pH) and/or sodium nitrate in 15 mM increments. Induction of S25DH_1_ production was achieved with 1 mM isopropyl β-D-1-thiogalactopyranoside (IPTG) at OD_578 nm_ ≈ 0.8 during exponential growth. When the cultures reached the stationary growth phase, the medium was no more supplemented, and the cultures were stored for up to three weeks at 30 °C or 4 °C.

### Determination of dissolved dioxygen

The concentration of dissolved oxygen in aqueous solutions was determined by non-invasive optical detection using SP-Pst3-D5 sensor spots (PreSens, Regensburg, Germany) immersed in the solution and a Fibox 4 trace oxygen meter (PreSens).

### Fluorescence microscopy of cell suspensions

A commercially available LIVE/DEAD™ BacLight™ Bacterial Viability Kit (Thermo Fisher Scientific, Waltham, USA) was used to investigate living/dead cells. *T. aromatica* cells were diluted to OD_578 nm_ = 1.0 in dH_2_O. The cell suspension (10 µL) was mixed with 10 µL of a staining solution (SYTO 9/ propidium iodide 1:1 [v/v]), incubated for 15 min at room temperature in the dark and analysed by fluorescence microscopy (Axio Imager.M2, Carl Zeiss, Oberkochen, Germany). GFP and dsRed filters were used for the LIVE/DEAD staining analysis at a 100x magnification.

### VitD_3_ conversion by whole cells

If not otherwise stated, VitD_3_ conversion assays were usually carried out in the 250 or 500 µL scale in Eppendorf tubes. Assay mixtures contained 0–10% (w/v) HPCD, 0.5 mM VitD_3_ (from a stock solution prepared in isopropanol), electron acceptors (0–10 mM K_3_[Fe(CN)_6_], 2 mM NaNO_3_ or O_2_ [shaking at 500 rpm using an Eppendorf ThermoMixer^®^]), S25DH_1_-producing *T. aromatica* cell suspensions at OD_578 nm_ 10–100 and a phosphate-buffered medium (5 mM NaH_2_PO_4_, 32 mM K_2_HPO_4_, 10 mM NH_4_Cl at pH 8.0). The densities of *T. aromatica* cell suspensions were adjusted by centrifugation in 50 mL conical tubes (6000 rpm, 4 °C, 20 min, Eppendorf 5804R centrifuge, Rotor S-4-72) and by resuspension in the required volume of phosphate-buffered medium. For conversion under anaerobic conditions, all steps were carried out with anaerobic buffers and solutions in a forming gas atmosphere (N_2_/H_2_, 95:5). VitD_3_ conversion assays were carried out for up to 24 h and sampled regularly by transferring an aliquot of the reaction mix (20 µL) into a fourfold volume excess of isopropanol (80 µL) to precipitate enzymes and cells. Product formation was detected by ultra-performance liquid chromatography (UPLC) measurements.

For whole cell conversion in the 100 mL scale, a premixed, saturated VitD_3_ solution (≈ 9.5 mM) was mixed in a 1:1 ratio (v/v) with a cell suspension at OD_578 nm_ = 200 (total reaction volume: 100 mL) and incubated at 30 °C for up to 50 h. The resulting reaction mix contained 4.63 mM of dissolved VitD_3_ in the presence of 12.5% (w/v) HPCD and 5% (v/v) ethanol. At the beginning and after 2 h, 4 h, 22 h and 28 h, 10 mM K_3_[Fe(CN)_6_] were added to result in a final concentration of 50 mM. The experiment was carried out for 50 h under anoxic conditions to ensure stability of the whole-cell catalyst. Samples were taken at defined time points and analysed by UPLC.

### VitD_3_ conversion by cell free extracts

A cell suspension with OD_578 nm_ = 200 was disrupted after addition of DNase I (Applichem, Darmstadt, Germany) by single passage through a French pressure cell (1100 psi, SLM Aminco) to prepare cell free extracts. Protein content was determined by Bradford [31], and the final protein concentration of the diluted extract in the reaction mixture (100 µL) was 2.8 mg mL^–1^. The setup for the VitD_3_ conversion including sampling procedure was as described for whole cells with 10% (w/v) HPCD, 10 mM K_3_[Fe(CN)_6_] and 0.5 mM VitD_3_.

### Preparation of premixed aqueous solutions saturated with VitD_3_

A solution of 50% (w/v) HPCD in ddH_2_O was prepared and mixed with the required volume of 100–200 mM VitD_3_ stock solution in ethanol to obtain a final concentration of 10–20 mM of VitD_3_ under constant stirring. The mixture was diluted with phosphate-buffered medium to the desired final volume. Approximately 50% of the added VitD_3_ precipitated and was removed by filtration (0.2 μm filter) to give a clear solution. Typical concentrations of 6–10 mM dissolved VitD_3_ (as determined by UPLC analysis) were obtained in the presence of 20–25% (w/v) HPCD and 5–10% (v/v) ethanol as co-solvent.

### Determination of malate dehydrogenase activity

Malate dehydrogenase activity was determined in cell free extracts and whole cells as an indicator of membrane integrity. NADH and oxaloacetate (250 µM, each) served as substrates in a 0.1 M potassium phosphate buffered (pH 7.4) environment (total volume: 0.5 mL). NADH oxidation was monitored time-dependently at 340 nm (ε_340 nm_ = 6.22 mM^–1^ cm^–1^) and 30 °C using a UV-1900i UV-Vis spectrophotometer (Shimadzu Corporation, Kyoto, Japan). Samples of whole cells or cell free extracts (5% [v/v] of the reaction volume) were diluted accordingly to ensure evaluable temporal absorbance changes.

### UPLC based analysis of VitD_3_ and 25OHVitD_3_

The conversion of VitD_3_ to 25OHVitD_3_ was monitored by UPLC analysis. After two centrifugation steps (20 min, RT, 14,000 rpm), samples (injection volume: 5 µL) were analysed using a Waters H-Class UPLC System (Waters, Milford, USA). Separation was achieved by reversed-phase chromatography (Waters Acquity UPLC CSH Fluoro-Phenyl column, 2.1 × 100 mm, 1.7 μm particle size) using an isocratic gradient of 65% acetonitrile + 0.1% (v/v) formic acid for 4 min at a flow rate of 0.4 mL min^–1^ and 50 °C column temperature. Detection of steroids was achieved by absorption measurements at 260 nm using a diode array detector. For determination of absolute VitD_3_ and 25OHVitD_3_ concentrations, peaks at 260 nm were integrated and compared to peak areas of authentic standards (Sigma-Aldrich, Taufkirchen, Germany). The correlation between peak area and VitD_3_/25OHVitD_3_ concentrations was linear in a range between 10 and 500 µM. If necessary, samples were diluted in isopropanol accordingly.

### Removal of cells and steroids for HPCD recycling

After conversion of VitD_3_, cells were removed from the reaction mixture by centrifugation (6,000 rpm, 4 °C, 20 min) and the product as well as residual VitD_3_ were extracted three times with a three-fold excess of ethyl acetate (v/v). To the remaining aqueous phase containing only HPCD and buffer components, fresh buffer was added to make up the original volume. This HPCD solution was used for the next cycle of bioconversion.

### Quantitative HPCD determination

The photometric determination of HPCD was modified from Goel et al. [32] and Mäkelä et al. [33] and was based on the decrease in phenolphthalein absorption after complexation with cyclodextrin. A working solution was prepared by mixing 20 mL of 125 mM NaHCO_3_ with 800 µL ethanol and 200 µL of a 4 mM solution of phenolphthalein in ethanol. Centrifuged HPCD samples (14,000 rpm, RT, 20 min) were diluted with 50 mM TRIS/HCl pH 7.0 to an HPCD concentration of 0-100 µg mL^–1^. The diluted samples (200 µL) were then mixed with 800 µL of working solution and the absorption at 550 nm was measured. For quantification, samples containing 0–50 µg mL^–1^ of HPCD were used for a calibration curve.

### Software used and statistical analysis

Graphics shown in this work were generated using OriginPro 2023 (OriginLab Corporation, Northampton, USA) and GraphPad Prism 6 (GraphPad Software, Boston, USA). Molecular formulae shown in various figures were drawn using ChemDraw 22 (PerkinElmer Informatics, Waltham, USA). UPLC data were acquired and analysed using Empower 3 (Waters, Milford, USA). Time-dependent absorption measurements were analysed using LabSolutions UV-Vis software (Shimadzu Corporation, Kyoto, Japan). Fluorescence microscopic images were acquired, merged and edited using ZEN Microscopy software (Carl Zeiss, Oberkochen, Germany). Results of technical replicate experiments (*n* = 2 or 3) are presented as mean ± standard deviation.

## Results

### Initial setup for the whole-cell conversion of VitD_3_ to 25OHVitD_3_ by S25DH_1_-producing *T. aromatica* resting cells

Using the recently established heterologous production platform for S25DH_1_ in *T. aromatica* (*T. aromatica* pIZ1016_S25dCBAD), extracts from cells grown with benzoate and nitrate as carbon and energy sources converted 1 mM VitD_3_ (0.385 g L^–1^) to 25OHVitD_3_ in ≈ 2 h with a yield of 90% [29]. In these assays, 10 mM K_3_[Fe(CN)_6_] was used as electron acceptor. Here, we aimed to use this platform for the whole-cell conversion of VitD_3_ to 25OHVitD_3_ by coupling periplasmic S25DH_1_ either to the endogenous respiratory chain of *T. aromatica* via cytochrome c or to external electron acceptors. To control and optimise all relevant parameters, we opted for a resting cell approach in which pre-cultured *T. aromatica* cells producing S25DH_1_ were concentrated to a high-density suspension in a phosphate-buffered medium without supplements. In initial setups, *T. aromatica* was grown under denitrifying conditions in which benzoate was replaced by acetate. With continuously fed acetate and nitrate (molar 1:1 ratio), an optical density at 578 nm (OD_578 nm_) of 3–5 was achieved under nitrate limitation and permanent pH control; gene expression was induced by IPTG addition at OD_578 nm_ ≈ 0.8. Cells were washed with medium without acetate/nitrate, concentrated to OD_578 nm_ ≈ 50 (≈ 16 mg cell dry weight per mL corresponding to ≈ 8 mg protein per mL) and supplemented with 5% (w/v) HPCD and varying electron acceptors. The reaction was initiated by the addition of 0.5 mM VitD_3_ and stopped with isopropanol, in which substrate and product were extracted.

**Fig. 2:**
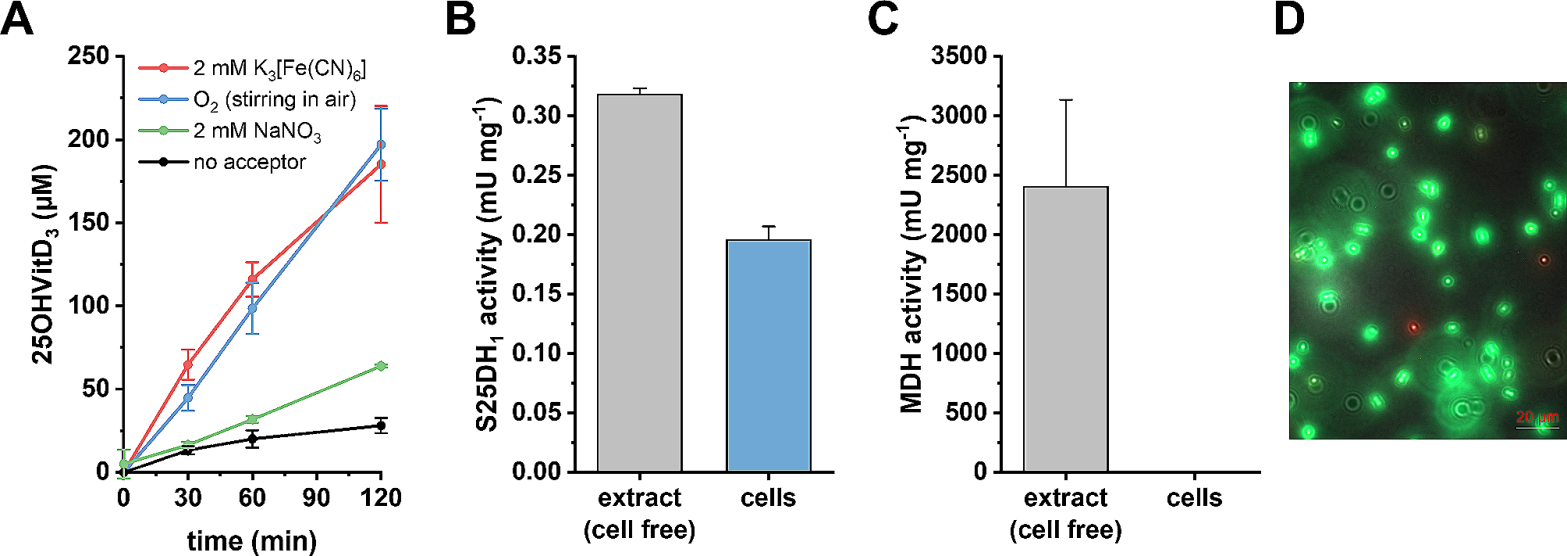
Electron acceptor-dependent whole-cell conversion of 500 µM VitD_3_ to 25OHVitD_3_ by recombinant *T. aromatica* cells producing S25DH_1_. (**A**), Electron acceptor dependence (0.25 mL scale, OD 50 corresponding to 4 mg cells [dry weight], 5% [w/v] HPCD, 1% [v/v] isopropanol). (**B**), S25DH_1_ activity in cell free extracts and whole cells (0.1 mL scale, 0.1–0.3 mg total protein [whole cell or cell extract] 10% [w/v] HPCD, 1% [w/v] isopropanol, 10 mM K_3_[Fe(CN)_6_]); 1 mU refers to 1 nmol min^–1^. (**C**), Malate dehydrogenase (MDH) activity in cell free extracts and whole cells; 1 mU refers to 1 nmol min^–1^. (**D**), Fluorescence microscopy visualizing the ratio of living (green) and dead (red) cells

The whole cell suspension converted VitD_3_ to 25OHVitD_3_ with the highest rates being observed in the presence of K_3_[Fe(CN)_6_] (2 mM) or in air with an initial rate of about 0.2 nmol (mg protein)^–1^ min^–1^ (Fig. 2A). When nitrate (2 mM) was used as acceptor under anoxic conditions, the conversion rate was only about 25%; the residual activity in the control without added electron acceptor can be attributed to residual endogenous electron acceptors. In a control experiment, isolated and enriched S25DH_1_ did not use O_2_ as direct electron acceptor, indicating that the observed O_2_-dependent conversion in whole cells depends on the endogenous electron transfer chain including a terminal oxidase. The S25DH_1_ activity per mg total protein in whole cells was approximately 60% of that in cell-free extracts (Fig. 2B). In contrast, cytoplasmic malate dehydrogenase activity was detected exclusively in cell-free extracts (Fig. 2C). This result indicates the integrity of the cells and excludes that S25DH_1_ activity originates from extracts of lysed cells. In consistence with this finding, fluorescence microscopy showed that approximately 90% of cells used in the whole cell conversion of VitD_3_ were viable (Fig. 2D).

We then tested the effect of the cell density on the VitD_3_ conversion rate (Fig. S1). By incrementally increasing the OD_578 nm_ of the cell suspension from 10 to 50, an expected increase in the VitD_3_ conversion rate was observed. A further doubling of OD_578 nm_ from 50 to 100 resulted only in a slight further increase of the overall VitD_3_ conversion rate. Unless not otherwise stated, whole cell conversion experiments were henceforth performed with suspensions at OD_578 nm_ = 50 (OD 50).

During whole-cell mediated VitD_3_ bioconversion, ultra-performance liquid chromatography (UPLC) analysis of samples taken at different time points during the whole-cell VitD_3_ conversion assays showed that 25OHVitD_3_ is the only hydroxylated product, whereas not even traces of calcitriol or other trihydroxylated compounds were found (Fig. S2). Very minor peaks (maximum 1% within 24 h) are attributed to thermal/photochemical isomerisation products [34].

### Optimized cultivation and stability of S25DH_1_-producing *T. aromatica*

To optimise the growth yield of S25DH_1_-producing *T. aromatica* cells, we tested different electron acceptors in combination with acetate as carbon and energy source. To ensure that the Moco biosynthetic machinery is upregulated [25, 29], nitrate was always added to the medium to serve either as an electron acceptor or as the sole nitrogen source. Notably, S25DH_1_ as well as the dissimilatory and assimilatory nitrate reductases all belong to the bis-molybdopterin dinucleotide cofactor-containing DMSO reductase enzyme family [25]. Therefore, the Moco biosynthetic machinery of S25DH_1_ and nitrate reductases should be induced under the growth conditions used to ensure sufficient levels of the common cofactor [35].

**Fig. 3:**
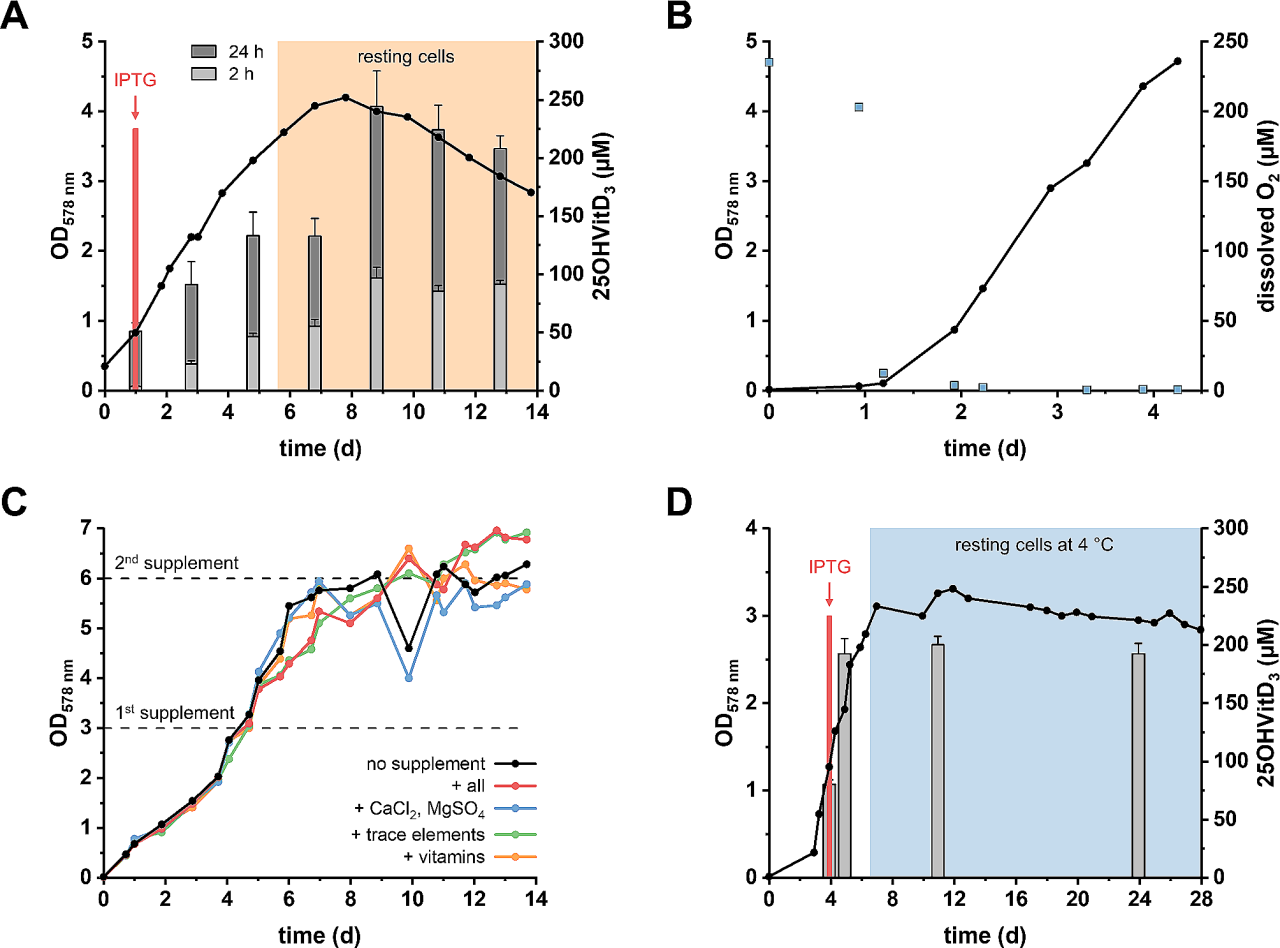
Semi-aerobic cultivation of *T. aromatica* producing S25DH_1_ with nitrate as electron acceptor. (**A**), Representative growth curve of *T. aromatica* producing S25DH_1_ with acetate + nitrate (1:1) under aerobic conditions without stirring/shaking at 30 °C (semi-aerobic, black line), and formation of 25OHVitD_3_ from 500 µM VitD_3_ (grey bars) by cells taken at different time points (0.5 mL scale, OD 50 corresponding to 8 mg cells [dry weight], 5% [w/v] HPCD, 1% [v/v] isopropanol, aerobic). (**B**), Representative growth curve under semi-aerobic conditions (black line) and oxygen consumption (blue squares). (**C**), Growth curve to maximal OD_578 nm_ and the effect of supplementation as indicated by the horizontal dashed lines. (**D**), Stability of *T. aromatica* cells producing S25DH_1_. Cells were grown under aerobic conditions + nitrate at 30 °C. After consumption of acetate/nitrate, the resting cells were stored at 4 °C; OD_578 nm_ (black line); 25OHVitD_3_ formation within 2 h from 500 µM VitD_3_ (grey bars) by cells taken at different time points (0.5 mL scale, OD 100 corresponding to 16 mg cells [dry weight], 5% [w/v] HPCD, 1% [v/v] isopropanol, aerobic)

Fully aerobic cultivation under continuous agitation in air resulted in maximum OD_578 nm_ values above 8 within 5 to 7 days, but the VitD_3_ conversion rate in OD 50 cell suspensions dropped below 10% (Fig. S3) compared to OD 50 suspensions prepared from cells grown with acetate/nitrate. This finding is assigned to the reported oxygen sensitivity of S25DH_1_ [25]. We therefore cultivated *T. aromatica* without stirring/shaking in aerobically prepared acetate/nitrate medium, referred to as semi-aerobic cultivation (Fig. 3A). In OD 50 suspensions of these cells, the conversion of VitD_3_ was comparable to suspensions from cells anaerobically grown with acetate/nitrate. Notably, the conversion was significantly higher in resting than in exponentially growing cells, although the OD_578 nm_ of the resting cells decreased after two days of incubation at 30 °C. This finding suggests that there was an ongoing S25DH_1_ synthesis in resting cells, and that S25DH_1_ remained active under the semi-aerobic cultivation conditions. Indeed, the oxygen was readily consumed by *T aromatica* during the first 24 h, before a significant increase of OD_578 nm_ was observed (Fig. 3B). This observation is consistent with the finding that cells grown under denitrifying conditions use oxygen as a preferred electron acceptor during VitD_3_ hydroxylation in *T. aromatica* cells and suggests that a terminal oxidase, most probably a low-affinity cytochrome *cbb*_*3*_-type oxidase, is produced under denitrifying conditions [36]. In summary, oxygen removal by the endogenous aerobic respiratory chain was sufficient to maintain S25DH_1_ activity if cells were not actively shaken or stirred. In further experiments to increase the maximal OD_578 nm_, different supplements were added. Indeed, supplementation with standard stock solutions of trace elements, vitamins or Ca^2+^, Mg^2+^ [30] at OD_578 nm_ 3 resulted in a maximum OD_578 nm_ of 6.5. However, this value could not be further increased, neither by a second supplementation (Fig. 3C), nor by suspending washed cells in fresh medium.

At 30 °C, resting *T. aromatica* cells began to lyse after 2–3 days (Fig. 3A). Cell lysis was increased in dense suspensions above OD 10. At 4 °C, the OD of cells (OD_578 nm_ 3–4) and the VitD_3_ conversion rate remained stable for up to three weeks under the previously defined semi-aerobic conditions (Fig. 3D). In conclusion, cell batches grown under semi-aerobic conditions were stored at 4 °C at OD_578 nm_ < 10, which facilitated further optimisation of whole-cell 25OHVitD_3_ synthesis in identical cell batches.

### Optimized solubilisation of VitD_3_

Like other isoprenoid side-chain containing steroids, VitD_3_ is almost insoluble in aqueous solutions. To overcome this problem in biocatalytic conversions of VitD_3_, HPCD has often been used to increase its solubility by several orders of magnitude up to the millimolar range [37]. In addition, HPCD promotes the isomerization of VitD_3_ to preVitD_3_, which is proposed to be the actual substrate for S25DH_1_ [28, 38].

**Fig. 4:**
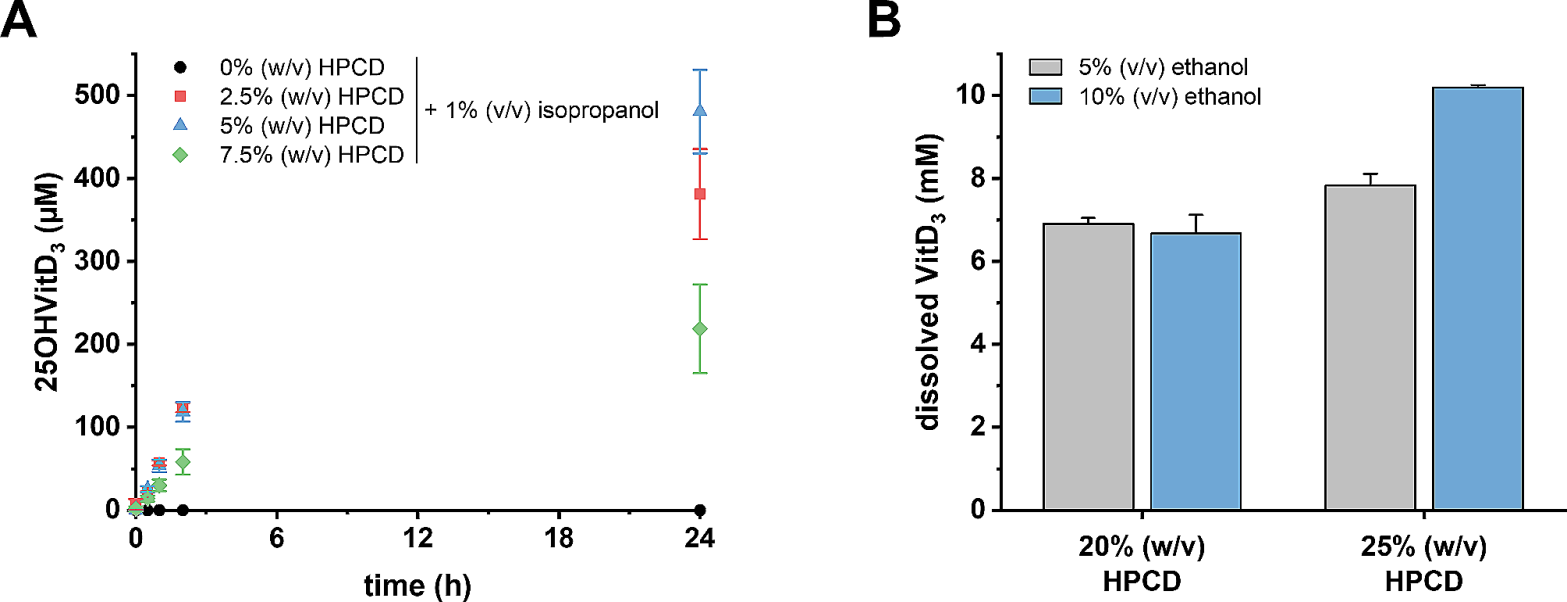
Effect of HPCD and isopropanol on whole-cell conversion and solubilisation of VitD_3_. (**A**), Effect of different HPCD concentrations on the formation of 25OHVitD_3_ from 500 µM VitD_3_ (0.25 mL scale, OD 50 corresponding to 4 mg cells [dry weight], 1% [v/v] isopropanol, aerobic). (**B**), Experimentally determined VitD_3_ concentrations in aqueous buffer using ethanol as co-solvent and HPCD

The dependence of whole-cell conversions of VitD_3_ to 25OHVitD_3_ on HPCD concentration was tested. In initial experiments, VitD_3_ stock solutions were prepared in isopropanol, from which aliquots were transferred to the medium resulting in a maximum of 0.5 mM dissolved VitD_3_ with 1% (v/v) isopropanol. In the absence of HPCD, virtually no formation of 25OHVitD_3_ from 0.5 mM VitD_3_ was observed (Fig. 4A). The optimum HPCD concentration for the conversion of 0.5 mM VitD_3_ was 5% (w/v), whereas at 7.5% (w/v), the conversion rate decreased. As reported for cell-free extracts [28], HPCD could not be substituted by organic solvents alone (isopropanol or ethanol, 1–10% [v/v], each) and only to a minor extent (< 20% VitD_3_ conversion compared to 5% [w/v] HPCD) by detergents (Tween 20, Triton X-100, or N,N-dimethyldodecylamine N-oxide, 0.2% each).

To maximise the amount of VitD_3_ solubilised in HPCD and co-solvent during the whole-cell conversion, stock solutions of 50% (w/v) HPCD in water and VitD_3_ solutions (20–200 mM) in either isopropanol or ethanol (1–10% [v/v]) were mixed at different ratios and the amount of solubilised VitD_3_ was determined by UPLC analysis. Under optimised conditions, up to 10 mM VitD_3_ were dissolved in the presence of 10% (v/v) ethanol and 25% (w/v) HPCD in aqueous buffer (Fig. 4B). These concentrated stock solutions were then used for whole cell suspension experiments. The activity of cells was not affected at final isopropanol/ethanol concentrations of up to 5% (v/v) in the reaction buffer. However, no VitD_3_ conversion was observed when HPCD was omitted in the presence of 5% (v/v) ethanol or isopropanol, respectively.

### Whole-cell 25OHVitD_3_ synthesis under optimized and upscaled conditions

**Fig. 5:**
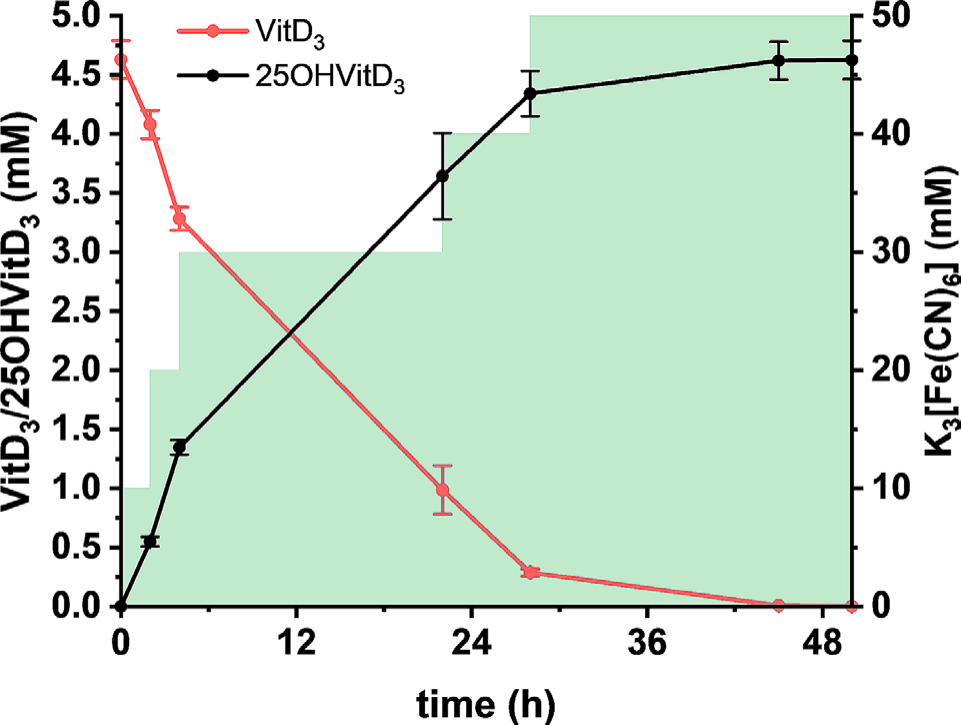
Time course of optimised conversion of 4.63 mM VitD_3_ to 25OHVitD_3_ using S25DH_1_-producing resting *T. aromatica* cells (100 mL scale, OD 100 corresponding to 3.2 g cells [dry weight], 12.5% [w/v] HPCD, 5% [v/v] ethanol, 50 mM K_3_[Fe(CN)_6_]). Electron-accepting K_3_[Fe(CN)_6_] was added in 10 mM increments (green shading)

The optimised conditions for cell cultivation, VitD_3_ solubilisation, and whole-cell conversion were applied to maximise biocatalytic conversion of VitD_3_ to 25OHVitD_3_. In addition, we scaled up the volume from the typical 0.5 mL to the 100 mL scale. For this purpose, an OD 200 resting cell suspension was mixed with an aqueous solution containing ≈ 9.5 mM VitD_3_, which was prepared in the presence of 25% (w/v) HPCD and 10% (v/v) ethanol as described above, resulting in a final concentration of 4.63 mM (1.78 g L^–1^) VitD_3_. To avoid inactivation of S25DH_1_ due to oxygen exposure, we chose K_3_[Fe(CN)_6_] as electron acceptor under anoxic conditions. In order to avoid conversion limitations due to electron acceptor consumption, K_3_[Fe(CN)_6_] was added in 10 mM increments to a total concentration of 50 mM over the course of the 50 h incubation. Using this setup, a titer of ≈ 3.8 mM (1.52 g L^–1^) 25OHVitD_3_ was achieved in the first 24 h, corresponding to 82% conversion. After prolonged incubation for further 26 h, the titer was increased to 1.85 g L^–1^ (4.6 mM) 25OHVitD_3_ corresponding to a > 99% conversion (Fig. 5). Note that the apparent higher titer of the product compared to the substrate is due to the higher molecular weight.

### Recycling of HPCD

The presence of HPCD is essential for an effective VitD_3_ hydroxylation by whole-cell systems [37], and the high concentrations required significantly increase the overall cost of biocatalytic 25OHVitD_3_ synthesis. To overcome this limitation, we aimed to develop a recycling process for HPCD for its use in multiple cycles of VitD_3_ bioconversion.

**Fig. 6:**
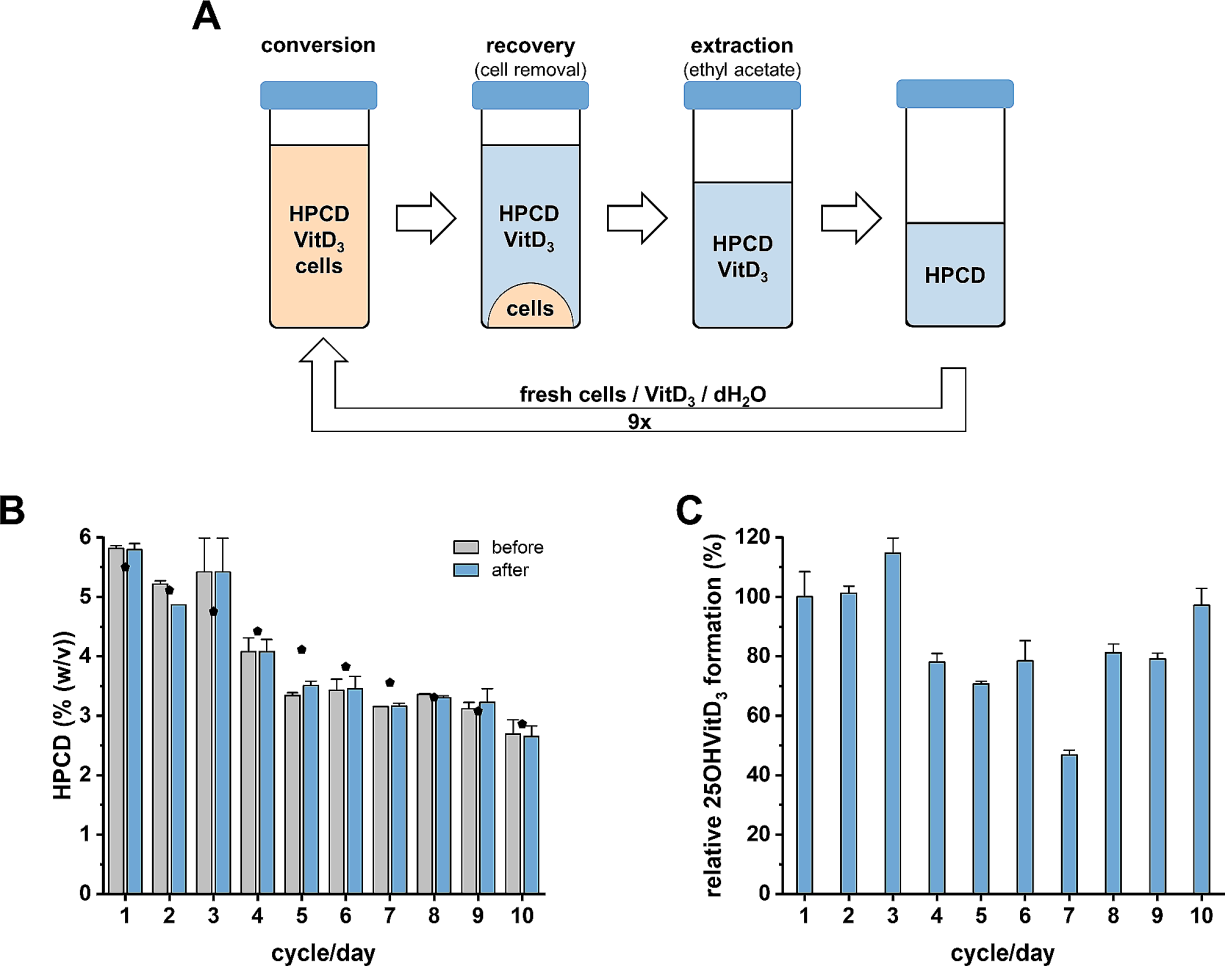
HPCD recovery. (**A**), General recycling setup. (**B**), Experimentally determined HPCD concentrations before and after ten consecutive VitD_3_ conversions. The black symbols represent the maximum expected HPCD concentration taking into account a 7% loss due to sampling and removal of cells and product. (**C**), Relative product formation in samples with freshly added HPCD (5% [w/v]) vs. recycled HPCD in ten consecutive VitD_3_ conversions within 4 h (0.5 mL scale, OD 100 corresponding to 16 mg cells [dry weight], 1% [v/v] isopropanol, aerobic). 100% refer to the respective formation of 25OHVitD_3_ with freshly added HPCD in each cycle

For HPCD recovery after VitD_3_ conversion, cells were removed by centrifugation and unconverted VitD_3_, the product 25OHVitD_3_ as well as the co-solvent isopropanol were extracted with ethyl acetate. The remaining aqueous phase contained HPCD and buffer components to which dH_2_O was added to the initial volume after each cycle to compensate for the loss of medium during cell removal and ethyl acetate extraction (Fig. 6A). For the ten consecutive reaction cycles tested, fresh cell suspensions (OD 100) were used for each cycle. In control experiments, the identical cell suspension charge to which new medium with freshly prepared HPCD was used for each cycle. During each cycle, a loss of approximately 6–7% of HPCD was estimated in samples with recycled and with freshly added HPCD, due to sampling for HPCD analysis and centrifugation/extraction procedures in the laboratory scale. This loss was the same in samples with recycled or freshly added HPCD, indicating that HPCD was not degraded during the ten VitD_3_ conversion cycles (Fig. 6B). The relative product formation was compared in samples with recycled vs. freshly added HPCD. Due to loss of HPCD after each cycle, a slight decrease of activity was expected in the samples using the recycled HPCD after each cycle. Although some variation was observed from cycle to cycle, the overall product formation indicates that recycled HPCD can replace freshly added HPCD (Fig. 6C). In conclusion, the established HPCD recycling process allows for multiple consecutive cycles of VitD_3_ conversion.

## Discussion

In this work, we have established a whole-cell system for the bioconversion of VitD_3_ to 25OHVitD_3_ that has several advantages over conventional oxygenase-/peroxygenase-based systems with respect to the product titer, yield and selectivity. With 1.85 g 25OHVitD_3_ L^–1^, the highest titer published was achieved with an almost complete conversion. Most importantly, the system hydroxylates VitD_3_ in the desired C25-position with >> 99% selectivity and does not produce even traces of the active hormone calcitriol.

**Fig. 7:**
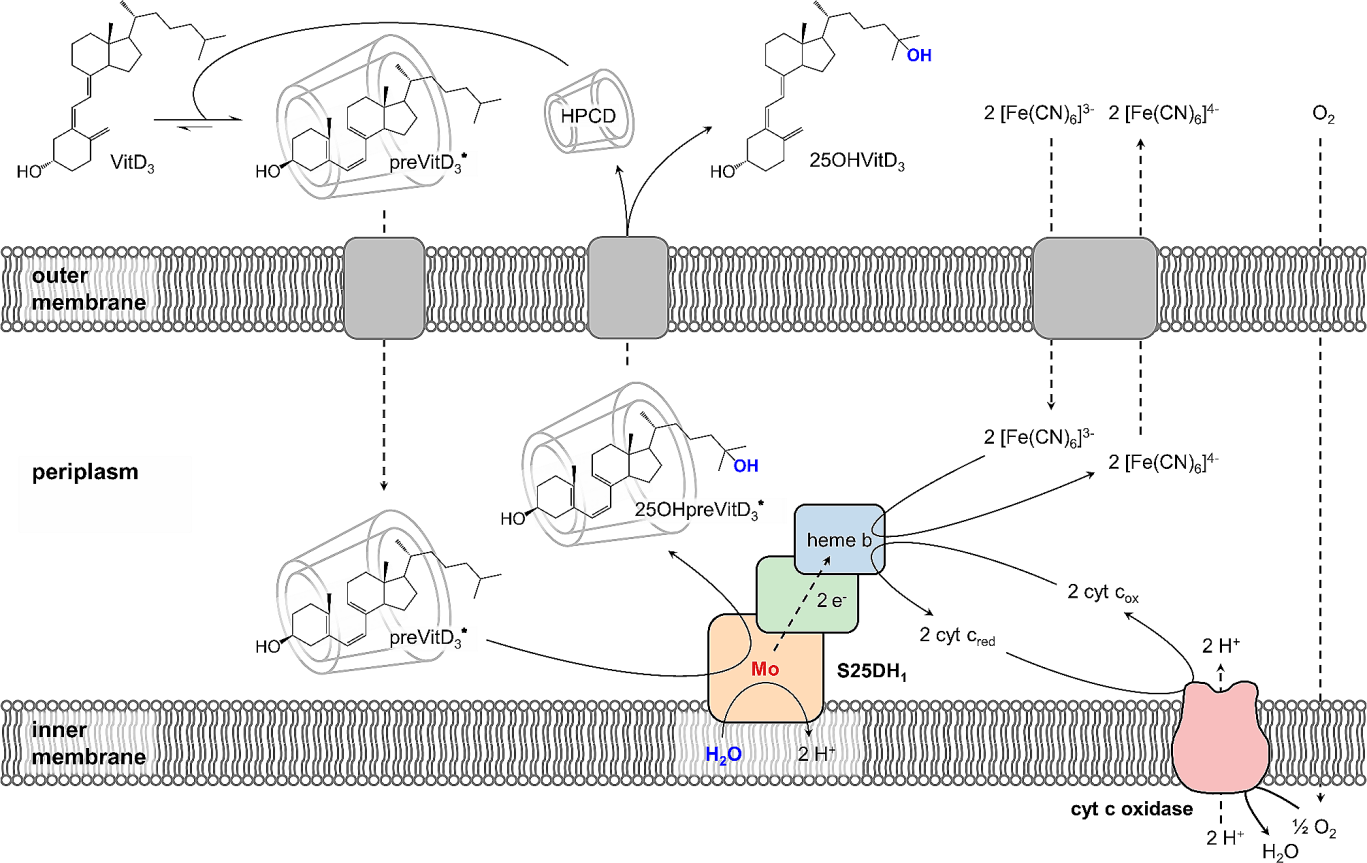
Proposed model for the whole-cell synthesis of VitD_3_ in S25DH_1_-producing *T. aromatica*. The preVitD_3_ form is favoured in the presence of HPCD and proposed to be the actual substrate of S25DH_1_. It is unclear whether the preVitD_3_/HPCD complex can pass the outer membrane through porins

The system using resting *T. aromatica* cells producing periplasmic S25DH_1_ from *S. denitrificans* fulfils a number of mandatory requirements for an efficient whole-cell 25OHVitD_3_ synthesis (Fig. 7). Firstly, VitD_3_ has to be taken up from the medium and transported across the outer membrane to reach periplasmic S25DH_1_. This process is not trivial, as VitD_3_ in the medium is tightly bound to HPCD forming a ≈ 2.85 kDa complex, from which it is unknown whether it can pass through an aqueous porin channel of the outer membrane from *T. aromatica*. However, considering that preVitD_3_ rather than VitD_3_ is the actual substrate of S25DH_1_ [28], and that the equilibrium between both isomers is significantly shifted to preVitD_3_ in the presence of HPCD [38], we assume that the VitD_3_/HPCD complex does indeed pass the outer membrane. The transport of cyclodextrin across a porin has been demonstrated in a *Klebsiella* sp [39]. Regardless of whether VitD_3_ in complex with HPCD passes the outer membrane, there will be an equilibrium between preVitD_3_ and VitD_3_ bound to cyclodextrin, to the outer and cytoplasmic membranes, and to the hydrophobic active site cavity of S25DH_1_. In this context, it is important to offer an excess of HPCD in order to keep the vast majority of VitD_3_/25OHVitD_3_ in the medium. Once VitD_3_ reaches the active site of S25DH_1_, the hydroxylation of VitD_3_ to 25OHVitD_3_ depends on an electron acceptor provided either by the endogenous O_2_-/nitrate-dependent respiratory chains or by the artificial K_3_[Fe(CN)_6_]. Although O_2_ and K_3_[Fe(CN)_6_] gave comparable conversion rates under optimal agitation, the latter is preferred due to the oxygen sensitivity of S25DH_1_. Oxygen damage to S25DH_1_ becomes increasingly problematic when cells are used for multiple cycles. With nitrate, the conversion rate was significantly lower. It is noteworthy, that the S25DH_1_ reaction generates electrons at a redox potential of the cytochrome c (>> +200 mV) rather than of quinone (+ 100 mV) level. Thus, a periplasmic nitrate reductase (NAP-type) could directly link VitD_3_ hydroxylation to cytochrome c oxidation, probably in a rather unspecific manner. Such a scenario would explain the significantly lower rates with nitrate compared to O_2_ or K_3_[Fe(CN)_6_]. NAP-type nitrate reductases can be induced in the stationary phase [40], which is in agreement with our observation that only resting but not exponentially growing cells accepted nitrate as an acceptor for VitD_3_ hydroxylation. Irrespective of the acceptor used, it was important to use resting cells in the absence of an additional electron acceptor. This finding can be rationalised by a competition between VitD_3_ and acetate (via its complete oxidation through the TCA cycle) as electron donors for K_3_[Fe(CN)_6_] or O_2_ reduction. After conversion, the hydroxylated product most likely crosses the outer membrane in the HPCD-bound state.

**Fig. 8:**
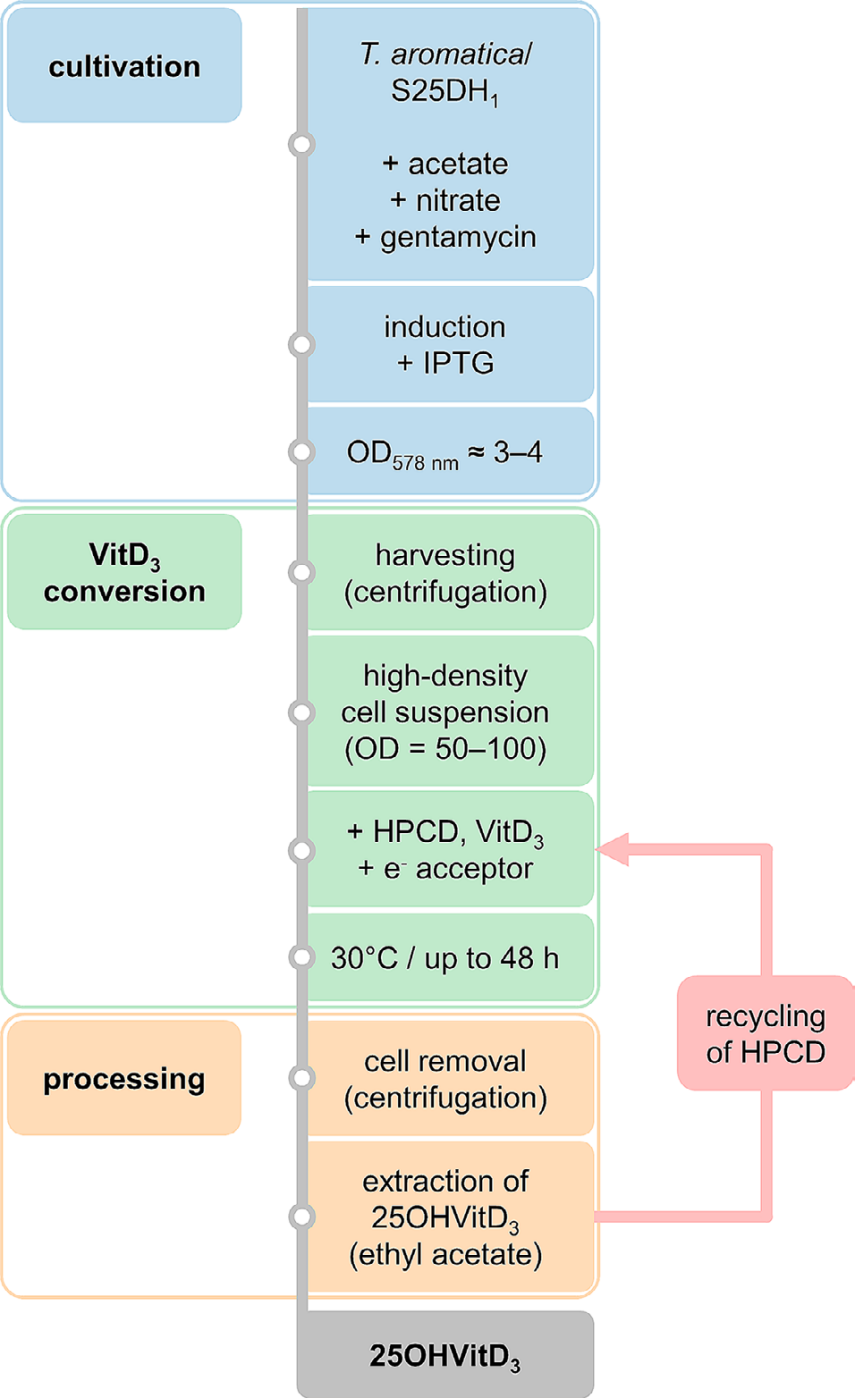
Workflow for whole-cell 25OHVitD_3_ biosynthesis using S25DH_1_-producing *T. aromatica*

The workflow of the established whole-cell 25OHVitD_3_ synthesis system can be divided into three main steps: (i) cultivation of the biocatalyst, (ii) conversion of VitD_3_ and (iii) processing of products and solvents (Fig. 8). Optimised cultivation was achieved under semi-aerobic conditions without stirring. Under these conditions, the O_2_ concentration was sufficient to act as an acceptor but low enough to cause no significant inactivation of S25DH_1_. Although we cannot exclude that some loss of S25DH_1_ activity occurred, the semi-aerobic conditions turned out to be optimal to facilitate the overall bioconversion process. To stimulate the entire Moco biosynthetic machinery for the active site cofactor of S25DH_1_, nitrate was always added to induce Moco-containing nitrate reductases [41]. It remains unclear why the growth of *T. aromatica* did not exceed OD_578 nm_ of 6.5 (semi-anaerobic) or 8 (aerobic), since even the exchange of medium did not allow higher cell densities to be achieved. On the other hand, the resting cells used for VitD_3_ conversion were stable for weeks when kept at low temperatures. This finding allows the three steps of cultivation, conversion and processing to be carried out separately and continuously, which will be of great advantage for future applications.

In conclusion, despite the slower cultivation time of *T. aromatica* compared to established whole-cell systems (e.g. *E. coli*), the C25 dehydrogenase based system for the biocatalytic conversion of VitD_3_ to 25OHVitD_3_ offers a number of advantages over previously reported biocatalytic systems. In order to scale up the process, a number of optimisations will need to be made in the future, in particular an increase in cell density and a better control of gene induction, ideally combined with the integration of the S25DH_1_ genes into the genome under the control of an endogenous promoter.

### Electronic supplementary material

Below is the link to the electronic supplementary material.

Supplementary Material 1

## Data Availability

Data is provided within the manuscript or supplementary information file. Further data including source data are available from the corresponding author on reasonable request.

## Abbreviations

CYP: Cytochrome P450
(d)dH_2_O: (Double) distilled water
DMSO: Dimethylsulfoxid
[Fe(CN)_6_]^3/4−^: Ferricyanide
HPCD: 2-hydroxypropyl-β-cyclodextrin
IPTG: Isopropyl β-D-1-thiogalactopyranoside
MDH: Malate dehydrogenase
Mo-*bis*PGD: Molybdo-*bis*-pyranopterin guanine dinucleotide
Moco: Molybdenum cofactor
NAP: Periplasmic nitrate reductase
OD_(578 nm)_: Optical density (at 578 nm)
25OHVitD_(3)_: 25-hydroxyvitamin D_(3)_
S25DH_(1)_: Steroid C25 dehydrogenase (isoform 1)
UPLC: Ultra-performance liquid chromatography
VitD_(2/3)_: Vitamin D_(2/3)_
