# Supplementary material for: Highly selective whole-cell 25-hydroxyvitamin D3 synthesis using molybdenum-dependent C25-steroid dehydrogenase and cyclodextrin recycling
Source: Microb Cell Fact. 2024 Jan 20;23:30. doi: 10.1186/s12934-024-02303-6 (PMC10799449; doi:10.1186/s12934-024-02303-6)
Supplement: Supplementary file 1 — Supplementary Material 1 [file 12934_2024_2303_MOESM1_ESM.docx]

Highly selective whole-cell 25-hydroxyvitamin D_3_ synthesis using molybdenum-dependent C25-steroid dehydrogenase and cyclodextrin recycling

Dennis Kosian^1,§^, Max Willistein^1,§^, Ralf Weßbecher^1^, Constantin Eggers^1^, Oliver May^2^, Matthias Boll^1,^*

^1^Faculty of Biology – Microbiology, University of Freiburg, 79104 Freiburg, Germany

^2^DSM Nutritional Products, Koninklijke DSM N.V., 4303 Kaiseraugst, Switzerland

Supplemental Information

Supplemental Figures 1–3


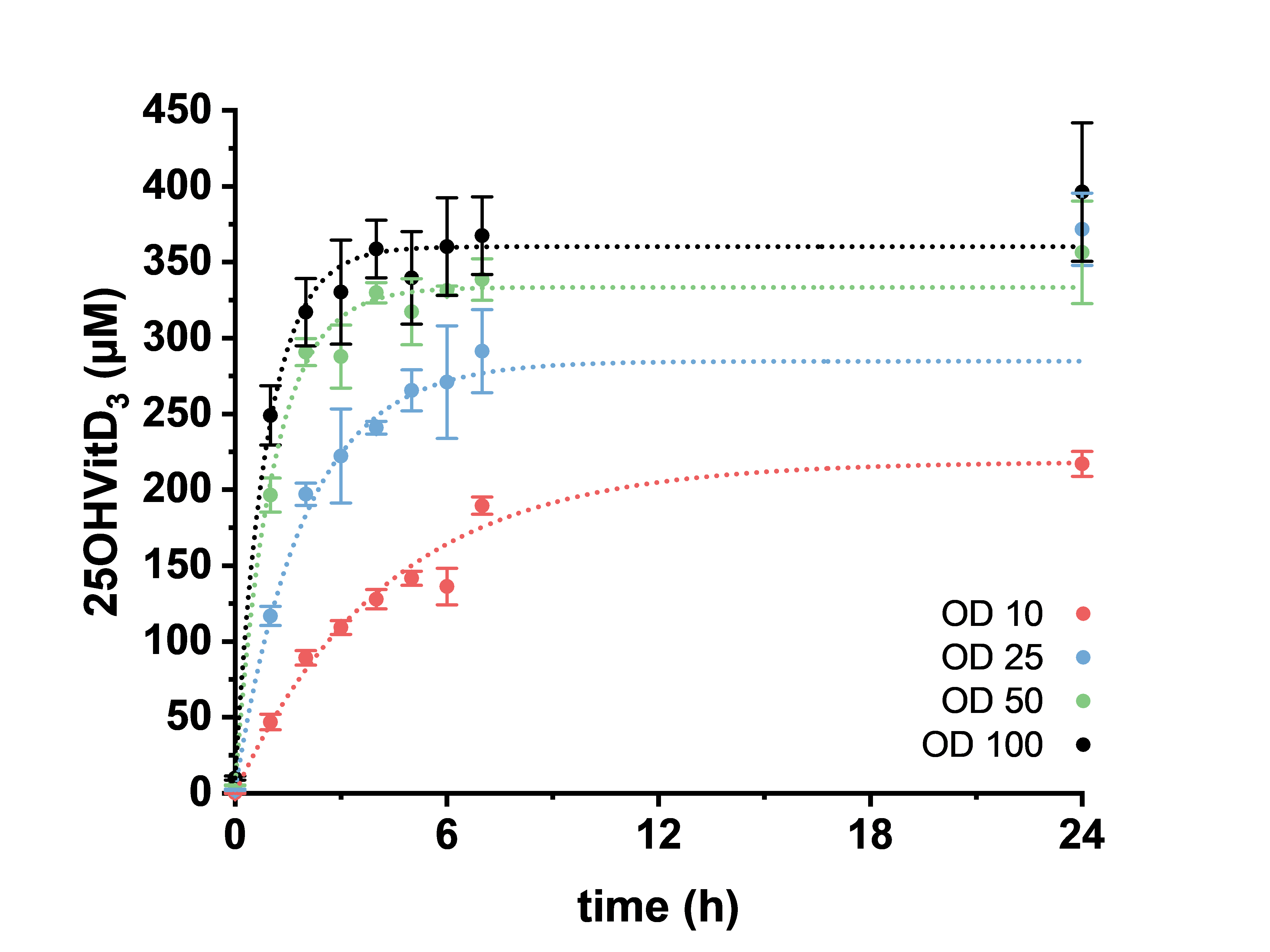


**Fig. S1. Effect of cell suspension density (OD_578 nm_) on 25OHVitD_3_ formation from 500 µM VitD_3_ conversion (0.25 mL scale, 5% [w/v] HPCD, 1% [v/v] isopropanol, aerobic).**


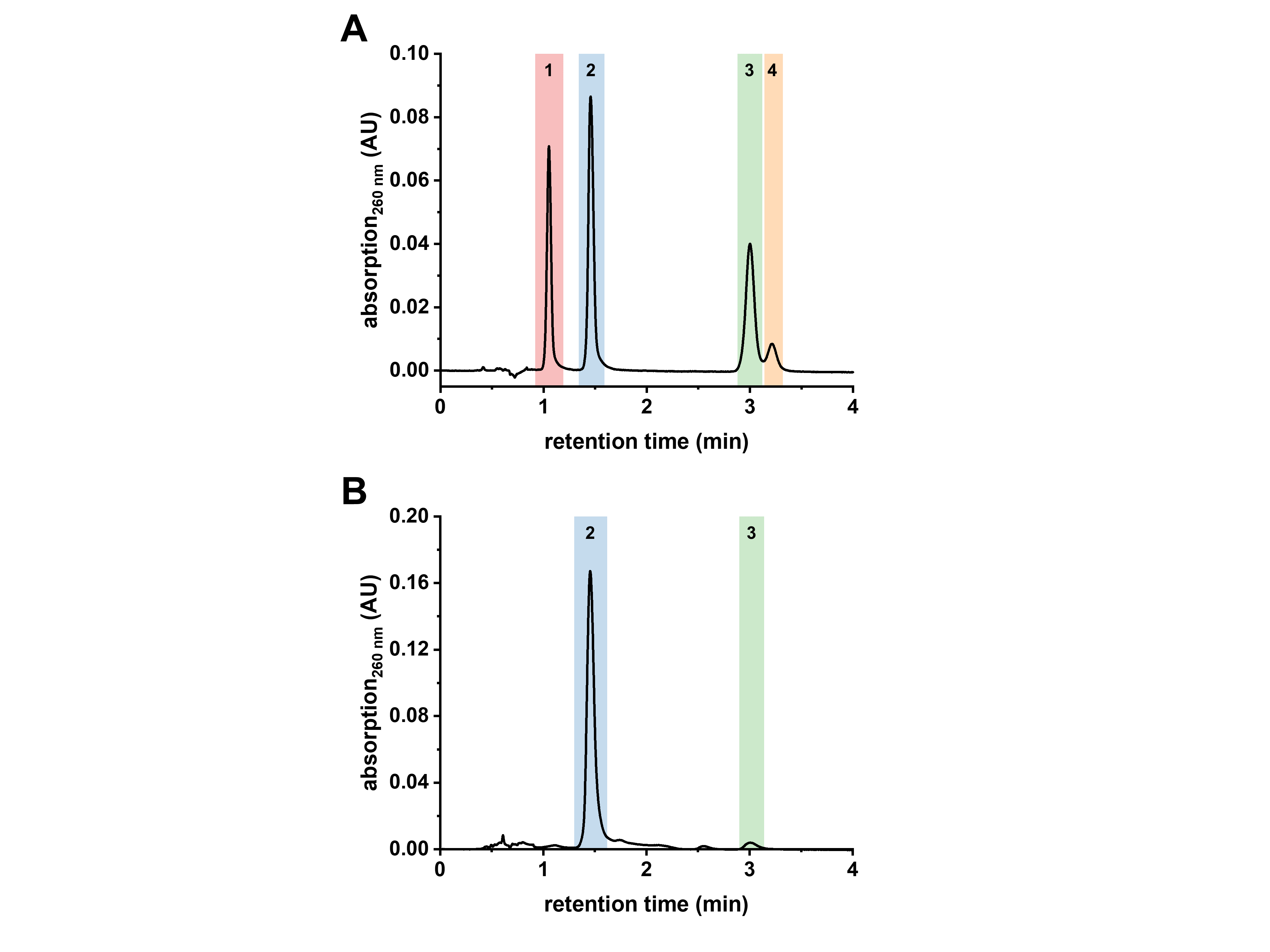


**Fig. S2. Comparison of Ultra Performance Liquid Chromatography (UPLC) chromatograms of authentic steroid standards (A, 50 µM each) with products formed during the conversion of 500 µM VitD_3_ with S25DH_1_-producing *T. aromatica* cells (B, after 20 h, 0.5 mL scale, OD 50 corresponding to 8 mg cells [dry weight], 5% [w/v] HPCD, 1% [v/v] isopropanol, aerobic). 1**, 1α,25(OH)_2_VitD_3_; **2**, 25OHVitD_3_; **3**, VitD_3_; **4**, 7-dehydrocholesterol.


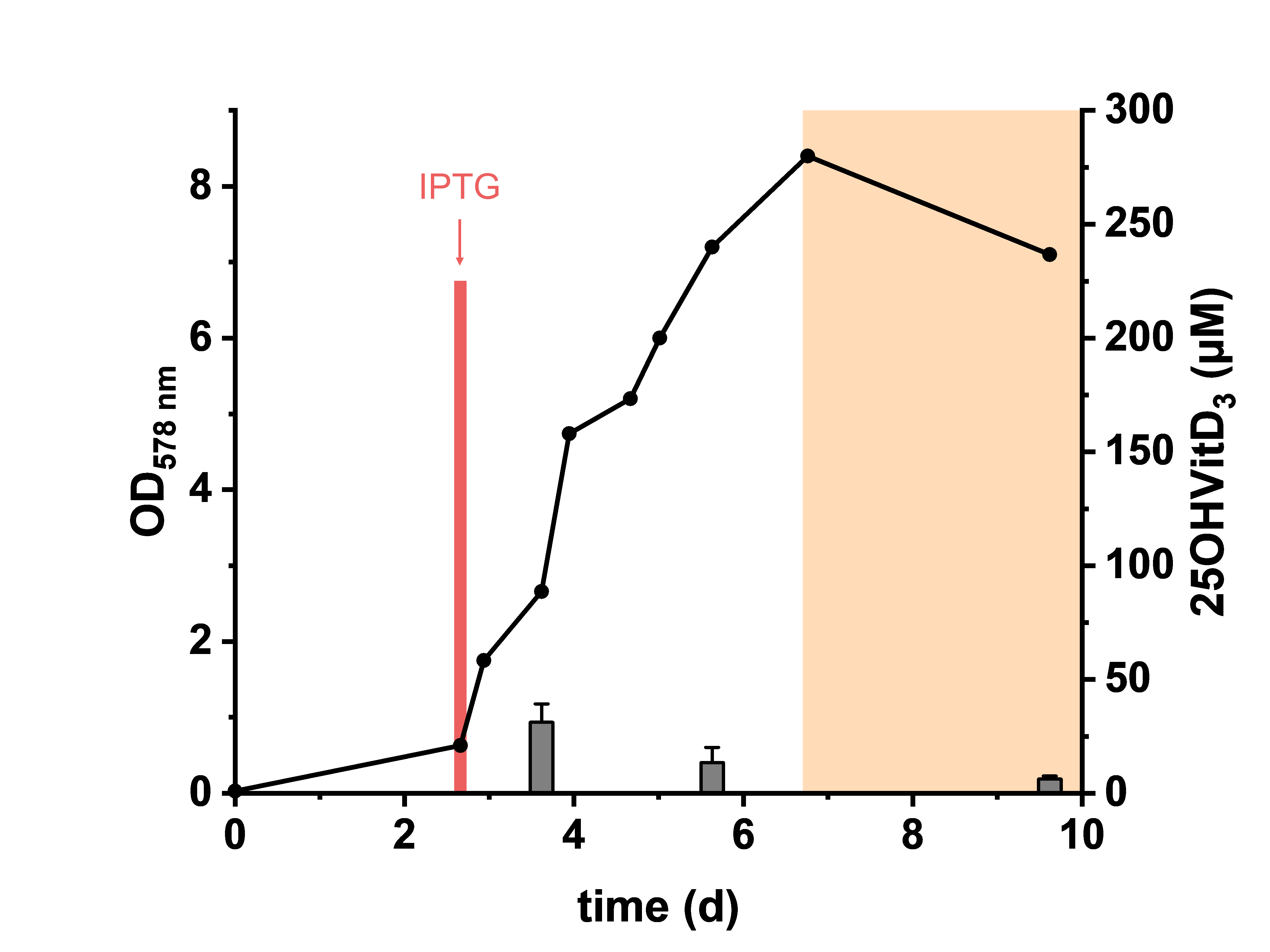


**Fig. S3. Growth curve of *T. aromatica* producing S25DH_1_ with acetate under fully aerobic conditions**. Black lines/circles: growth curve; grey bars: 25OHVitD_3_ formation from 0.5 mM VitD_3_ within 20 h from cells taken at different time points as indicated (0.5 mL scale, OD 50 corresponding to 8 mg cells [dry weight], 5% [w/v] HPCD, 1% [v/v] isopropanol, aerobic).
